# Diurnal Variations in Anterior and Posterior Corneal Thickness and Curvature in Healthy Eyes: Global and Sector-Based Metrics and Lifestyle Associations

**DOI:** 10.3390/life16050711

**Published:** 2026-04-22

**Authors:** Laura Barberán-Bernardos, Miguel Angel Ariza-Gracia, Philippe Büchler, David P. Piñero

**Affiliations:** 1 Department of Optics, Pharmacology and Anatomy, University of Alicante, 03690 Alicante, Spain; laura.barberan@ua.es; 2 ARTORG Center for Biomedical Engineering Research, University of Bern, 3008 Bern, Switzerland; miguel.ariza@unibe.ch (M.A.A.-G.); philippe.buechler@unibe.ch (P.B.); 3 Department of Ophthalmology (IMQO-Oftalmar), Vithas Medimar International Hospital, 03016 Alicante, Spain

**Keywords:** cornea, circadian rhythms, keratometry, corneal thickness, intraocular pressure, lifestyle factors

## Abstract

This study aims to characterize diurnal changes in corneal geometry of the anterior and posterior cornea in healthy eyes using global and sector-based metrics, and to explore their associations with lifestyle-related factors. A prospective observational study of 109 eyes was conducted, measuring intraocular pressure (IOP), corneal thickness, volume, and global/sector-based keratometry at five time points over a 10 h period, alongside a lifestyle questionnaire. Results revealed significant diurnal decreases in IOP, central and minimum corneal thickness, and corneal volume (*p* ≤ 0.031). While posterior keratometry remained stable, anterior keratometry showed statistically significant but clinically negligible changes (amplitude of change ≤ 0.29 D), primarily within the central 4 mm and inferotemporal regions. Weak to moderate positive correlations were found between water and coffee intake, smoking, and changes in corneal thickness and volume, with no relevant effects on keratometric parameters. Overall, corneal thickness and volume exhibit significant diurnal reductions, whereas anterior keratometric changes are minimal and clinically irrelevant globally. These findings underscore the importance of considering the time of day when interpreting corneal measurements, as lifestyle factors appear to modulate corneal thickness but not curvature in healthy eyes.

## 1. Introduction

The cornea contributes approximately two-thirds of the total refractive power of the eye [1]. Consequently, understanding its dynamic behavior is of significant relevance in clinical practice. Even subtle variations in corneal structure or geometry may affect refractive measurements and influence clinical decisions in settings such as refractive surgery planning, contact lens fitting, and intraocular lens power calculation [2,3]. A precise characterization of corneal stability over time is therefore crucial for the reliable interpretation of ocular measurements and for minimizing potential measurement-related biases.

Previous studies have demonstrated that the cornea is not static throughout the day but undergoes physiological fluctuations that can influence its properties. Specifically, intraocular pressure (IOP) and central corneal thickness (CCT) exhibit diurnal variations, with both parameters typically showing higher values during the early morning hours following awakening [4,5,6]. These changes are commonly attributed to nocturnal eyelid closure and reduced oxygen availability, which promote transient corneal edema [7]. As the day progresses and normal corneal metabolism and oxygenation are restored, this edema gradually resolves, leading to measurable changes in corneal thickness.

In contrast, the presence and magnitude of diurnal changes in corneal curvature remain less well established. While some authors have described statistically significant variations in anterior and posterior keratometry over the course of the day, others have reported minimal or no clinically relevant changes [8]. Specifically, a study evaluating the mean central corneal radius using Scheimpflug topography reported no significant diurnal changes [9]. In contrast, another study using a swept-source OCT system, reported a gradual flattening of the cornea over the course of the day, which was inversely correlated with IOP [10]. Conversely, Lau & Pye, using an autokeratometer described an initial steepening of the cornea in the early hours after awakening, followed by relative stability throughout the day [11]. This lack of consensus may be related to differences in measurement techniques, regional corneal analysis, sample characteristics, or control of factors such as time since awakening.

In this context, IOP represents a potential mechanical factor influencing corneal shape. Fluctuations in IOP change the biomechanical balance of the cornea, which leads to short-term changes in curvature or asphericity, particularly in regions with lower biomechanical resistance. However, the relationship between IOP, corneal geometry, and diurnal variation remains incompletely understood.

Lifestyle factors such as hydration, caffeine intake, and smoking have also been suggested to influence corneal properties. Hydration and caffeine have been reported to affect corneal biomechanics, both reducing corneal deformability [12,13]. Similarly, tobacco use has been associated with increased central corneal thickness and reduced endothelial cell density [14]. A previous study suggested that certain lifestyle habits may influence circadian rhythms at the scleral level, with breakfast intake reducing minimum sagittal height, coffee reducing mean sagittal height, and morning face washing reducing minimum bulbar slope [15]. Understanding the potential impact of these modifiable factors is therefore important when interpreting diurnal corneal variations and ensuring accurate assessment in both clinical and research settings.

The aim of this study was to characterize diurnal changes in corneal geometry (thickness and curvature) of the anterior and posterior cornea in healthy eyes using global and sector-based metrics, and to explore their associations with lifestyle-related factors; additionally, load-normalized curvature indices were derived to contextualize geometric changes with respect to intraocular pressure.

## 2. Materials and Methods

### 2.1. Study Population and Experimental Protocol

This study was conducted at the Optometric Clinic of the University of Alicante (Alicante, Spain). The study protocol was conducted in accordance with the principles of the Declaration of Helsinki and was approved by the Ethics Committee of the University of Alicante (UA-2023-01-19_2). All participants provided written informed consent prior to enrollment. Eligibility criteria comprised individuals over 18 years of age presenting a corrected distance visual acuity of 20/25 or better. Exclusion criteria included the presence of any ocular pathology or systemic disease, and a history of ocular surgery. To allow adequate corneal recovery before examination, contact lens wearers were required to suspend lens use for a minimum of 14 days for soft contact lenses, and at least 28 days for rigid gas permeable lenses.

All assessments were performed by the same examiner (L.B.B.) to ensure consistency. The examinations began with an initial assessment to determine eligibility for study inclusion. This included medical history, subjective and objective refraction, slit-lamp biomicroscopy, and ocular biometry using the IOLMaster 500 (Carl Zeiss Meditec, Jena, Germany).

The Visionix VX650 device (Visionix, Chartres, France) was used to measure IOP. Three consecutive readings were obtained for each eye, and the mean value was used for subsequent analysis. In addition, the Pentacam HR system (OCULUS Optikgeräte GmbH, Wetzlar, Germany) was employed to assess corneal volume, pachymetry, and anterior and posterior keratometry. Corneal volume was derived from a three-dimensional reconstruction of the corneal structure generated from the 25,000 elevation points acquired by the device and was calculated within a 10 mm diameter zone centered on the corneal vertex, considering both the anterior and posterior corneal surfaces as the limits for its estimation [16]. Measurements obtained with both the Visionix VX650 and the Pentacam HR were repeated at five different time points throughout the same day. Assessments were performed every 2.5 h, starting at 9:00 a.m. and continuing until 7:00 p.m. This schedule was designed to capture diurnal fluctuations in corneal parameters within a single day, covering a total period of 10 h.

**Figure 1:**
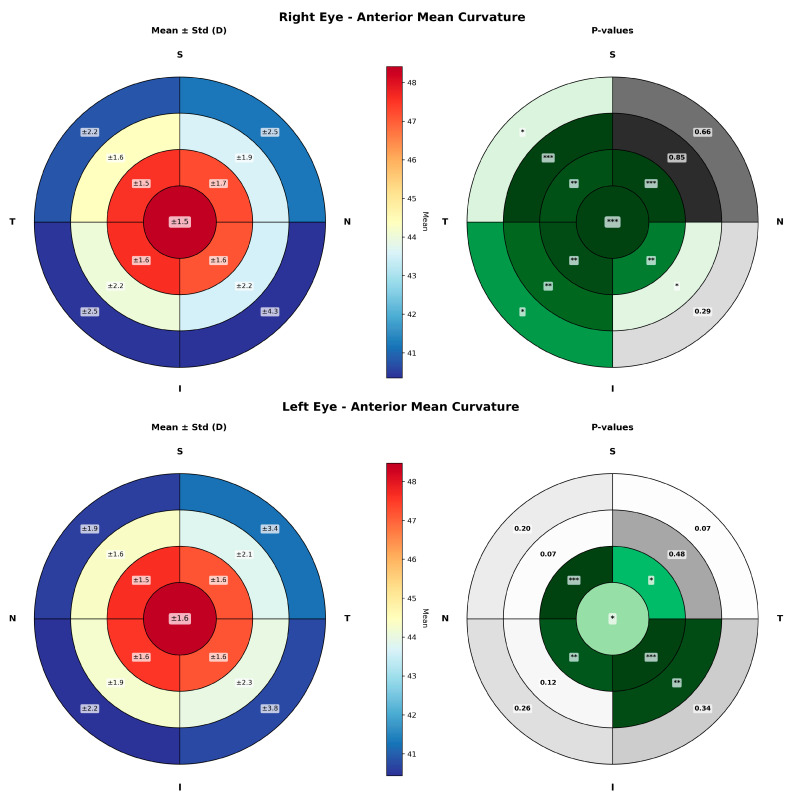
Sectorial maps of anterior corneal curvature showing diurnal changes across the corneal surface in right (**up**) and left eyes (**down**). For each eye, maps of mean ± standard deviation (**left**) and corresponding *p*-value (**right**) maps are presented. The cornea was divided into 1 mm radial rings and four sectors (nasal-superior, nasal-inferior, temporal-inferior, and temporal-superior). Asterisks denote the level of statistical significance: one asterisk for *p*-values between 0.05 and 0.01, two asterisks for *p*-values between 0.01 and 0.001, and three asterisks for *p*-values below 0.001. Green colours (the darker the colour, the lower the *p*-value associated) represent areas with statitically significant changes and grey colors (the darker the colour, the higher the *p*-value associated) are associated to those areas not showing significant changes.

Participants completed an ad hoc morning and evening questionnaire, described in a previous study [15] to record potential factors that could influence corneal measurements. The questionnaires were based on self-reported information and captured general lifestyle habits. The morning assessment captured information on sleep patterns, morning habits, ocular care, caffeine and water intake, and any physical activity performed early in the day. The evening assessment focused on daily lifestyle factors, including consumption of caffeine, alcohol, or tobacco, physical activity during the day, exposure to screens, naps, reading habits, and stressful events experienced. Figure 1 illustrates an overview of the procedure followed during the study.

### 2.2. Sample Size

The sample size was estimated based on previously reported variability and the expected magnitude of change. Consideration was also given to potential data loss during follow-up, as well as to maintaining appropriate statistical power and significance thresholds for hypothesis testing. A previously published study examining daily fluctuations in CCT in healthy subjects was used as the closest methodological reference [17]. Sample size estimation was performed using the online GRANMO calculator version 8.0 (IMIM, Barcelona, Spain), using CCT as the parameter of interest. Based on an assumed standard deviation of 0.008 mm for the amplitude of change, a minimum detectable difference of 0.0034 mm in central corneal thickness, an anticipated dropout rate of 20%, a statistical power of 80%, and a significance level (α) of 0.05, the required sample was determined to be 55 eyes.

### 2.3. Corneal Mean Curvature and Power

Mean curvature (*κ_H_* [m^−1^]; resp. mean power *H* [D]) was selected as the primary geometric descriptor because it provides an intrinsic, local measure of surface bending that is independent of coordinate system or reference axis [18]. It is derived directly from the two principal curvatures of the surface and therefore reflects local surface geometry in both meridional directions. This makes mean curvature well suited for quantitative analysis of corneal shapes that deviate from idealized rotational symmetry or exhibit localized deformation. In addition, mean curvature has direct physical relevance in the context of corneal tissue mechanics, as it relates to bending behavior in thin shells and biological surfaces.

Anterior corneal elevation data were obtained from corneal topography and represented as a continuous surface using a Zernike polynomial expansion defined over the analysis region. The elevation map was fitted with Zernike polynomials up to order 7, yielding a smooth, twice-differentiable surface (*z*(*x*,*y*)) suitable for analytic computation of spatial derivatives [19]. Mean curvature was then computed pointwise across the reconstructed corneal map using standard differential geometry expressions based on first- and second-order derivatives of the fitted surface.

(1) $$\kappa_{H}\left(x,y\right)=\frac{\left(1+z_{y}^{2}\right)z_{xx}-2z_{x}z_{y}z_{xy}+\left(1+z_{x}^{2}\right)z_{yy}}{2{\left(1+z_{x}^{2}+z_{y}^{2}\right)}^{3/2}}\;\left[m^{-1}\right],$$

where (*z_x_*,*z_y_*) and (*z_xx_*,*z_xy_*,*z_yy_*) are the first- and second-order derivatives of the Zernike surface.

Curvature values were converted to optical power assuming a spherical interface. For the anterior corneal surface, refractive indices of air (*n*_0_ = 1.000) and cornea (*n*_1_ = 1.376) were used, while for the posterior surface refractive indices of cornea (*n*_0_ = 1.376) and aqueous humor (*n*_1_ = 1.336) were assumed [20]. Optical power was computed from the local curvature based on the refractive index step across each surface.

(2) $$H\left(x,y\right)=\left(n_{1}-n_{0}\right)\kappa_{H}\left(x,y\right)\;\left[D\right].$$

In the following sections, the term mean curvature refers to the mean optical power (*H*, in diopters); geometric curvature (*κ_H_*, in mm^−1^) was not used for the primary analysis.

### 2.4. A Proxy for Corneal Biomechanics

To relate routinely measured clinical variables (IOP, thickness, and corneal curvature) to a biomechanically meaningful quantity, we used a thin-shell equilibrium framework in which the cornea is approximated as a pressurized membrane. Under quasi-static conditions, the normal force balance links pressure to in-plane membrane stress and local curvature through Young–Laplace/Laplace-type relations [21,22]. In an isotropic membrane approximation, the equilibrium can be written in terms of mean curvature H as:

(3) $$p=2\cdot \sigma_{iso}\left(x,y\right)\cdot t\left(x,y\right)\cdot \kappa_{H}\left(x,y\right),$$

where *p* is IOP [kPa], *t*(*x*,*y*) is local thickness [mm], *κ_H_* (*x*,*y*) is mean curvature [mm^−1^], and *σ_iso_* (*x*,*y*) is the corresponding in-plane membrane stress [kPa]. (Equivalently, in principal curvature directions one may write *p* = *t*(*x*,*y*)(*σ*_1_ · *κ*_1_ + *σ*_2_ · *κ*_2_), with *κ_H_* = (*κ*_1_ + *κ*_2_)/2.)

Because the goal of this study was not to estimate a material modulus but to obtain a load-normalized geometric response metric, we defined a pressure-normalized curvature coefficient *C_NORM_*. First, mean curvature and IOP were expressed relative to reference values representative of a typical cornea and typical IOP:

(4) $$H_{rel}\left(x,y\right)=\frac{H\left(x,y\right)}{H_{0}}\;\left[–\right],\;p_{rel}=\frac{p_{t}}{p_{0}}\;\left[–\right],$$

where *H*_0_ = 43 D [20,23,24] and *p*_0_ = 15 mmHg [25,26] are normal reference constants, and *p_t_* denotes the measured IOP at the corresponding time point. The coefficient was then computed as the ratio of relative curvature to relative pressure:

(5) $$C_{NORM}\left(x,y\right)=\frac{H_{rel}\left(x,y\right)}{p_{rel}}.$$

By construction, *C_NORM_* is dimensionless and increases when curvature is higher than expected for the measured pressure (and decreases when curvature is lower than expected). This coefficient is therefore interpreted as a proxy for deformation response per unit load, not as a direct estimate of intrinsic material stiffness.

### 2.5. Sector-Based Analysis of Corneal Curvature and Thickness

Regional analysis of corneal curvature and thickness was performed by partitioning the cornea into concentric annular regions centered on the corneal apex with radii of 0–1, 1–2, 2–3, and 3–4 mm. The central annulus (0–1 mm) was analyzed as a single region, whereas the peripheral annuli were further subdivided into nasal–superior, temporal–superior, temporal–inferior, and nasal–inferior sectors, in addition to the annular mean. For each eye, surface, visit, ring, and sector, mean values of mean curvature (H) and corneal thickness (T) were extracted from the corresponding maps.

For each regional metric, descriptive statistics were computed by pooling all available individual measurements across subjects and visits, yielding an overall mean and standard deviation. To evaluate whether regional values changed significantly over time, a non-parametric Friedman test was applied to repeated measurements across visits [27]. For this analysis, data were organized at the patient level, with one value per visit; only subjects with complete data across all five visits were included. A minimum of three complete subjects was required for the Friedman test to be performed, otherwise the corresponding *p*-value was reported as missing. All analyses were performed separately for each eye, surface, metric, ring, and sector.

### 2.6. Correlation Analysis and Heatmap Visualization

Pairwise associations between corneal, biometric, and behavioral variables were assessed using Kendall’s rank correlation coefficient (τ) [28]. Pairwise associations between corneal, biometric, and behavioral variables were assessed using Kendall’s rank correlation coefficient *p* < 0.05 were retained.

Visit-specific correlation matrices were visualized using heatmaps with non-significant correlations masked [29,30]. To summarize associations across visits, the number of visits with significant correlations was counted for each variable pair, and mean τ values were computed across significant visits only. These summary correlations were visualized using annotated heatmaps.

In addition, correlations were computed between behavioral variables and subject-level temporal features derived from repeated measurements, including robust trend slopes (Theil–Sen), Mann–Kendall trend statistics, first-to-last differences, peak-to-valley ranges, coefficients of variation, and lag-1 autocorrelation.

### 2.7. Statistical Analysis of Global Corneal Indices

Statistical analyses were performed on global corneal indices (i.e., spatially averaged, non–sector-based metrics) using SPSS software (version 29.0.1; IBM Corp., Chicago, IL, USA). The normality of the data distribution was assessed using the Kolmogorov–Smirnov test. For evaluation of diurnal changes in corneal parameters, repeated-measures analysis of variance (ANOVA) was used for parametric variables [31], while the non-parametric Friedman test was applied for variables that did not meet normality assumptions [32]. Post hoc pairwise comparisons were conducted with Bonferroni correction when significant differences were detected. Amplitude of change was defined as the difference between the maximum and minimum values recorded across the five timepoints. *p*-values < 0.05 were considered statistically significant.

## 3. Results

### 3.1. Sample Characteristics

A total of 109 eyes (55 right eyes and 54 left eyes) from 55 subjects were analyzed. The mean age of the study population was 32.6 ± 12.6 years, with ages ranging from 18 to 67 years. The sample consisted of 37 female and 18 male participants.

The mean spherical equivalent refractive error was −1.32 ± 2.31 diopters in right eyes (RE) and −1.19 ± 2.23 diopters in left eyes (LE), with mean visual acuity of −0.10 ± 0.05 logMAR in RE and −0.10 ± 0.07 logMAR in LE. Mean axial length was 23.90 ± 1.13 mm for and 23.89 ± 1.13 mm, for RE and LE respectively.

Participants reported an average sleep duration of 7.14 ± 1.04 h on the night preceding data acquisition. The time elapsed between awakening, and the first measurement was 2.08 ± 0.84 h on average, with an interquartile range of 1.58–2.47 h.

Regarding lifestyle and behavioral factors recorded through the ad hoc questionnaire, only a small proportion of participants (3.6%) reported a diagnosis of sleep apnea. Most participants (65.5%) had breakfast, and slightly over half (52.7%) drank water in the morning. Morning coffee consumption was reported by 56.4% of subjects, and the majority washed their face after waking (89.1%). Eye drop use in the morning was uncommon (7.3%), and approximately half of the participants rubbed their eyes (47.3%). Morning exercise was reported by 14.5% of participants. Most participants (83.9%) reported screen exposure before bedtime.

During the day, 63.6% consumed coffee, while 16.4% were current smokers. Daytime exercise was reported by 23.6% of participants. Alcohol consumption was rare (1.8%), and 25.5% experienced a stressful event. Napping during the day was uncommon (5.5%), and about half of the participants (49.1%) engaged in reading non-digital content.

### 3.2. Diurnal Changes in Corneal Geometry

Diurnal changes in IOP, keratometry and corneal thickness and volume are presented in Table 1. Statistically significant differences were observed in IOP, corneal thickness, and corneal volume, all of which decreased throughout the day (*p* ≤ 0.031). Anterior keratometry in the right eye showed a statistically significant, though clinically insignificant, increase over the course of the day in the right eye (*p* ≤ 0.005). Post hoc analysis with Bonferroni correction revealed significant differences between visits two and four for the anterior flattest keratometry (K1) (*p* = 0.023), and between visits two and four (*p* = 0.009) as well as visits one and four for the anterior steepest keratometry (K2) (*p* = 0.009). The remaining keratometry parameters remained stable.

Figure 1 shows the mean and standard deviation of anterior keratometry values across the different corneal regions, analyzed in 1 mm radial rings and divided into nasal-superior, nasal-inferior, temporal-inferior, and temporal-superior sectors. The corresponding *p*-value maps illustrate the spatial distribution of statistically significant changes over time. In the RE, statistically significant differences between measurement times throughout the day were observed, predominantly in the temporal-inferior sector. In the LE, significant changes were also detected in the temporal-inferior sector but concentrated in a more central region.

**Figure 2:**
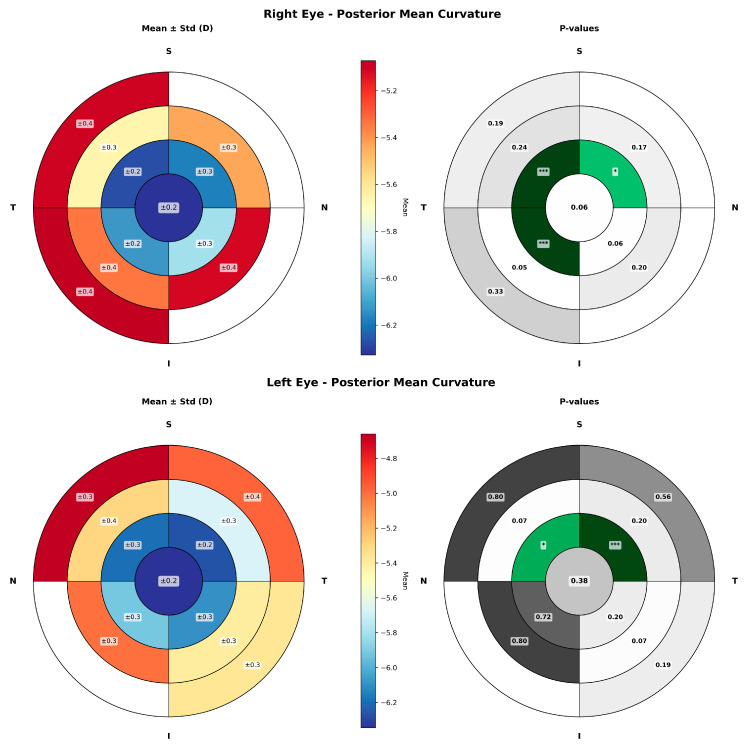
Sectorial maps of posterior corneal curvature showing diurnal changes across the corneal surface in right (**up**) and left eyes (**down**). For each eye, maps of mean ± standard deviation (**left**) and corresponding *p*-value (**right**) maps are presented. The cornea was divided into 1 mm radial rings and four sectors (nasal-superior, nasal-inferior, temporal-inferior, and temporal-superior). Asterisks denote the level of statistical significance: one asterisk for *p*-values between 0.05 and 0.01, and three asterisks for *p*-values below 0.001. Green colours (the darker the colour, the lower the *p*-value associated) represent areas with statitically significant changes and grey colors (the darker the colour, the higher the *p*-value associated) are associated to those areas not showing significant changes.

In Figure 2, posterior keratometry changes throughout the day in the different sectors are presented, revealing no statistically significant differences between measurement times in either eye, indicating stability of diurnal posterior keratometry.

**Figure 3:**
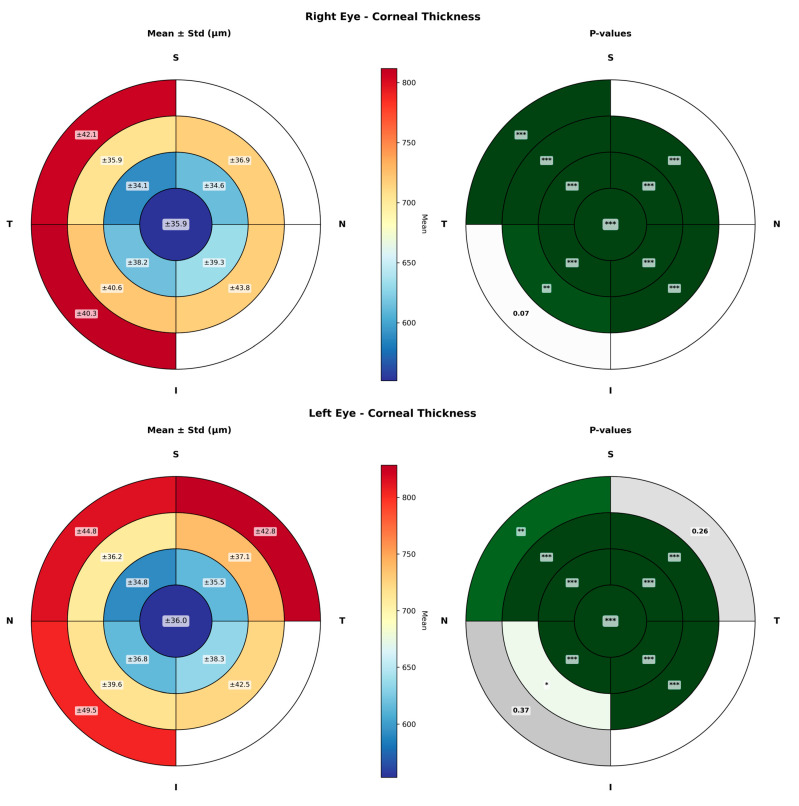
Sectorial maps of corneal thickness showing diurnal changes across the corneal surface in right (**up**) and left eyes (**down**). For each eye, maps of mean ± standard deviation (**right**) and corresponding *p*-value (**left**) maps are presented. The cornea was divided into 1 mm radial rings and four sectors (nasal-superior, nasal-inferior, temporal-inferior, and temporal-superior). Asterisks denote the level of statistical significance: one asterisk for *p*-values between 0.05 and 0.01, two asterisks for *p*-values between 0.01 and 0.001, and three asterisks for *p*-values below 0.001. Green colours (the darker the colour, the lower the *p*-value associated) represent areas with statitically significant changes and grey colors (the darker the colour, the higher the *p*-value associated) are associated to those areas not showing significant changes.

Figure 3 presents the corresponding analysis for corneal thickness. The mean and variability maps, together with the *p*-value maps, show statistically significant diurnal changes across nearly all sectors analyzed in RE and LE, with standard deviations reaching up to 49.5 μm.

### 3.3. Diurnal Changes in Corneal Aberrations and C_NORM_

The Zernike coefficients and the *C_NORM_* values for each of the five assessments throughout the day are presented in Table 2. Spherical aberration showed a significant increase in both eyes over the study period. In the RE, values increased from the first to the second and fifth assessments (*p* = 0.042, *p* = 0.019 respectively), while in the LE, a significant increase was observed between the first and fifth assessments (*p* = 0.007). In contrast, vertical secondary astigmatism exhibited a significant decrease over time, with reductions detected between the third and fifth assessments in RE (*p* = 0.010) and between the second and fifth assessments in LE (*p* = 0.014). *C_NORM_* values demonstrated a consistent upward trend in both eyes, with significant increases observed across multiple paired comparisons in RE (first vs. fifth, *p* = 0.007; second vs. fifth, *p* = 0.042; and third vs. fifth, *p* = 0.019) and LE (second vs. fourth, *p* = 0.059; second vs. fifth, *p* = 0.005; and third vs. fifth, *p* = 0.059).

### 3.4. Correlation Between Corneal Parameters and Lifestyle Habits

**Figure 4:**
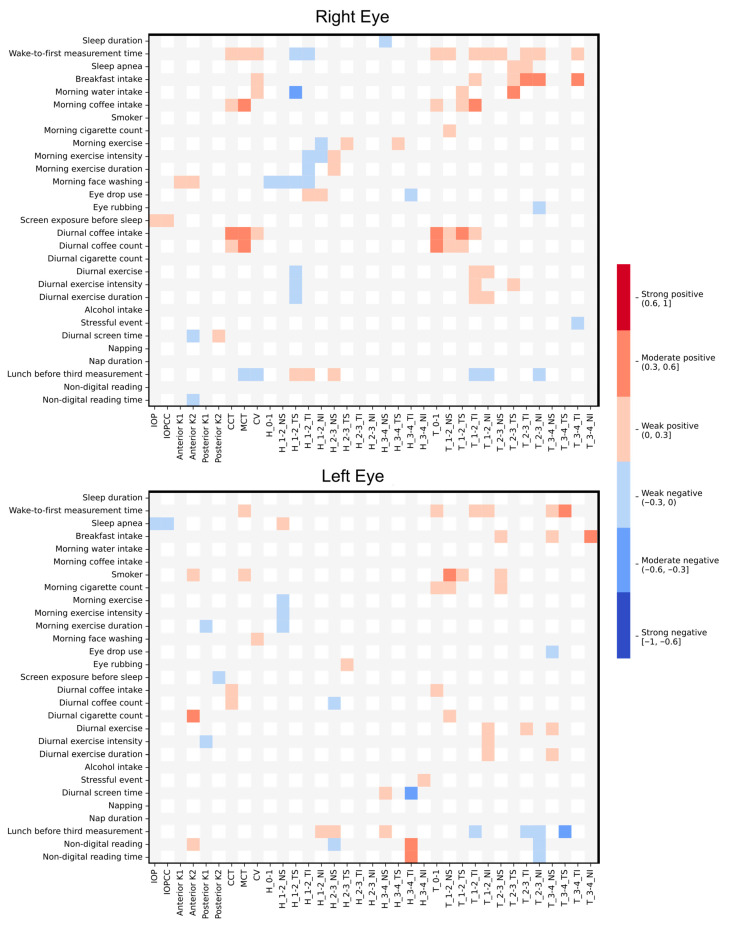
Heatmap of correlation coefficients between lifestyle habits and the change (first–to–last measurement difference) in corneal parameters. Warmer and cooler colors represent positive and negative correlations, respectively. Abbreviations: IOP = intraocular pressure; IOPcc = corneal-compensated intraocular pressure; CCT = central corneal thickness; MCT = minimum corneal thickness; CV = corneal volume; H_x-y = mean curvature in the ring between diameters x and y (mm); T_x-y = corneal thickness in the ring between diameters x and y (mm); NS = nasal–superior, TS = temporal–superior, TI = temporal–inferior; NI = nasal–inferior.

Figure 4 presents a heatmap illustrating the correlations between corneal parameters and lifestyle-related variables. Weak to moderate positive correlations were observed between both water and coffee intake and CCT, minimum corneal thickness (MCT), and corneal volume. Time elapsed since the first measurement showed positive correlations with corneal thickness across almost all analyzed corneal rings. Smoking status was also positively correlated, with weak to moderate strength, with corneal thickness within the central 3 mm radius. No relevant correlations were observed between lifestyle habits and keratometry parameters.

## 4. Discussion

Statistically significant diurnal variations were observed in both anterior keratometry and corneal thickness in healthy subjects. Specifically, corneal thickness and corneal volume decreased over the course of the day, consistent with progressive resolution of overnight corneal hydration effects. Although statistically significant variations were observed in the anterior K1 and K2 values, these changes remain clinically insignificant, with a maximum amplitude of change of 0.29 D. However, quadrant-based analysis reveals a non-uniform spatial distribution of keratometry changes. The magnitude of variation is predominantly concentrated in the central corneal region, particularly within the central 4 mm diameter zone, as well as in the inferotemporal areas.

From a clinical perspective, these findings highlight the importance of considering the time of day when performing corneal assessments, particularly in contexts requiring high measurement precision, such as refractive surgery planning, contact lens fitting, or monitoring of corneal disease progression. Although the observed diurnal variations were small, accounting for them may help reduce sources of measurement variability and improve consistency in longitudinal evaluations.

The diurnal behavior of anterior keratometry remains controversial in the peer-reviewed literature. While some studies have reported an increase in anterior keratometry during daytime hours, others have described a decrease or an absence of meaningful diurnal change in healthy eyes [8]. Nevertheless, the magnitude of the curvature changes reported is minimal, with variations in the radius of curvature generally on the order of 0.03 to 0.07 mm, which may be regarded as clinically insignificant [11,33]. These inconsistent findings indicate that the direction and magnitude of anterior keratometry variations are not uniformly defined and may depend on factors such as measurement timing, analytical methodology, and the specific parameters used to characterize corneal shape.

The anterior cornea was found to exhibit the largest changes in the central and inferotemporal sector. This localized behavior explains why analyses based on single global keratometry metrics, such as the flattest or steepest keratometric values, fail to capture these subtle alterations. By reducing keratometry to a single representative value, these changes can be masked. Despite the presence of statistically significant differences, measurement variability remains low, with standard deviations not exceeding 2.3 D in the central diameter 6 mm. These localized changes further emphasize that global keratometric indices may underestimate regional variability, which could be relevant in procedures requiring high precision, such as toric intraocular lens calculation or corneal refractive surgery planning.

In contrast, the posterior cornea showed no significant diurnal change, with a maximum amplitude of 0.11 D. This finding contrasts with a previous report, which described a steepening of the posterior cornea upon awakening, with mean changes in posterior corneal best fit sphere of 0.04 in the central region and 0.05 D in the peripheral region [17]. In that study, the same corneal topography system was used; however, the analyzed zones differed, as a central 3.5 mm diameter and a surrounding peripheral ring up to 7 mm were considered. Similarly, Biswas & Biswas reported, using a swept-source OCT system, an amplitude of change of 0.03 D and 0.02 D in anterior K1 and K2, respectively, with statistically significant differences observed only in K1 [10]. Differences in imaging technology, such as Scheimpflug-based systems and swept-source OCT, which rely on distinct acquisition principles and sampling strategies, may also influence the sensitivity of posterior corneal curvature measurements to subtle diurnal changes.

Regarding corneal thickness, both CCT and MCT showed a progressive decrease over the course of the day, with maximum mean reductions of 8.29 ± 4.24 µm for CCT and 9.04 ± 6.75 µm for MCT. Sector-based analysis indicated that this thinning pattern was relatively homogeneous across the entire corneal surface, with no marked regional predominance. These findings are consistent with those previously reported in some studies that showed a generalized diurnal reduction in corneal thickness in healthy eyes [6,34,35]. Specifically, a study by Read & Collins reported mean diurnal changes of 0.019 mm in the central cornea and 0.022 mm in the peripheral cornea, with the temporal sector showing a smaller amplitude of change compared with the superior and nasal peripheral sectors [17]. The results of the present study showed diurnal variations in pachymetry in most corneal sectors, with smaller changes observed in the peripheral regions compared with the central regions.

Diurnal variations in corneal higher-order aberrations were observed, with spherical aberration showing a small but significant increase over the day in both eyes, while vertical secondary astigmatism decreased. Consistent with these findings, Mierdel et al. also reported a diurnal increase in vertical secondary astigmatism, with a mean change of 0.016 µm, but no change in spherical aberration [36]. In contrast, Read et al. did not find significant diurnal differences in vertical secondary astigmatism in healthy eyes [37], which could be related to differences in the calculation of Zernike coefficients. *C_NORM_* increased significantly throughout the day, consistent with the observation that corneal curvature also became slightly steeper. This suggests an increased corneal deformation response to IOP later in the day, likely driven by diurnal changes in corneal shape and hydration.

A direct, although weak to moderate, correlation was observed between water and coffee intake and the diurnal change in CCT, MCT and corneal volume. These results suggest that hydration status and caffeine consumption may influence corneal thickness, potentially through osmotic or biomechanical mechanisms. A study by Redondo et al. [38] reported no significant effect of caffeine intake on CCT or anterior chamber depth, although they reported that caffeine increased IOP. Moreover, Jiménez et al. [13] demonstrated that caffeine consumption reduces corneal deformability, supporting the hypothesis that caffeine may alter corneal biomechanical behavior without necessarily inducing large absolute changes in thickness. However, a study analyzing changes in pachymetry after drinking water found no correlation between CCT and water intake [39]. The discrepancy between studies may be explained by differences in study design, timing of measurements after caffeine intake, or sensitivity of the instruments used to detect subtle corneal changes.

Time elapsed from awakening to the first morning measurement showed a consistent direct correlation with the variation in corneal thickness across nearly all corneal rings. This finding aligns with well-established evidence of diurnal corneal thinning following overnight hypoxia and eyelid closure, with gradual thinning occurring during waking hours as corneal metabolism and oxygenation normalize [7]. The persistence of this correlation across peripheral rings suggests that diurnal corneal changes are not limited to the central cornea but affect the cornea more globally. These results highlight the importance of controlling for time of day when assessing corneal parameters.

Smoking status was weakly to moderately positively correlated with the change in corneal thickness within the central 3 mm radius. This observation is consistent with the findings of Frifelt et al. [14], who reported an association between tobacco use and increased CCT. However, other studies have found no significant differences in CCT between smokers and non-smokers in healthy subjects [40,41]. These conflicting results may reflect differences in smoking intensity, duration of exposure, sample size, or population health status. It is also possible that smoking-related changes in corneal thickness are subtle and region-specific, which may explain why the present study detected correlations primarily in the central cornea.

Some inter-eye asymmetries were observed in the present study. A potential explanation for this finding could be habitual sleep position; however, this variable was not recorded in the study. It has been reported that sleep position may influence corneal biomechanics and contribute to ocular asymmetry in conditions such as keratoconus, potentially increasing biomechanical stress on the cornea [42]. In addition, sleep position has been associated with differences in ocular surface parameters and dry eye symptoms, with side sleeping showing higher symptom severity compared with supine positioning [43]. In healthy subjects, such effects are less well understood, and their influence on corneal diurnal behavior remains speculative. Future studies specifically designed to evaluate sleep posture and its relationship with corneal biomechanics and ocular surface parameters may help clarify this issue.

Several limitations of this study should be acknowledged. First, the sample size was relatively small and consisted of healthy young adults, which may limit the generalizability of the findings to older populations or patients with ocular pathology. Second, although lifestyle habits were recorded using an ad hoc questionnaire, self-reported data may be subject to recall bias. Finally, corneal measurements are algorithm-dependent estimates rather than direct physical measurements, as they rely on device-specific reconstruction models based on optical and mathematical assumptions.

## 5. Conclusions

Corneal parameters exhibit statistically significant diurnal variations in healthy subjects. While variations in anterior keratometry were statistically significant, they were clinically negligible, highlighting the importance of quadrant and sector-based analyses to detect subtle localized changes, with the most pronounced changes observed in central and inferotemporal corneal regions. Corneal volume and thickness, including both CCT and MCT, decreased progressively over the course of the day, following a relatively homogeneous pattern across the corneal surface, likely due to the reduction of overnight corneal edema. The observed weak to moderate associations between hydration, caffeine intake, smoking, and corneal thickness should be interpreted with caution, as they may reflect preliminary trends that warrant further investigation in larger and more precisely characterized samples. Overall, these findings emphasize the need to consider time of day and participant habits when interpreting corneal measurements in clinical and research settings.

## Figures and Tables

**Table 1:** Diurnal changes in intraocular pressure, anterior and posterior keratometry, corneal thickness, and corneal volume.

		09:00	11:30	14:00	16:30	19:00	Amplitude	*p*-Value
IOP (mmHg)	RE	16.36 ± 3.19	16.16 ± 2.62	15.96 ± 2.67	15.50 ± 2.72	15.33 ± 2.49	3.29 ± 1.72	<0.001 *
	LE	15.41 ± 2.28	15.77 ± 2.23	15.50 ± 2.44	14.83 ± 2.36	15.10 ± 2.62	3.03 ± 1.72	<0.001 *
IOPcc (mmHg)	RE	16.15 ± 2.93	15.96 ± 2.35	15.78 ± 2.53	15.35 ± 2.52	15.19 ± 2.42	3.29 ± 1.71	0.031 *
	LE	15.14 ± 2.12	15.45 ± 1.89	15.25 ± 2.32	14.56 ± 2.10	14.87 ± 2.54	3.05 ± 1.76	0.002 *
Anterior K1 (D)	RE	47.87 ± 1.53	47.89 ± 1.48	47.92 ± 1.51	47.96 ± 1.50	47.92 ± 1.50	0.28 ± 0.21	0.005 *
	LE	47.87 ± 1.58	47.86 ± 1.58	47.94 ± 1.59	47.90 ± 1.60	47.90 ± 1.62	0.26 ± 0.11	0.067
Anterior K2 (D)	RE	48.84 ± 1.69	48.87 ± 1.68	48.91 ± 1.73	48.93 ± 1.68	48.91 ± 1.68	0.29 ± 0.11	0.002 *
	LE	48.95 ± 1.80	48.93 ± 1.80	48.99 ± 1.78	48.96 ± 1.81	48.97 ± 1.82	0.28 ± 0.15	0.493
Posterior K1 (D)	RE	−6.16 ± 0.23	−6.15 ± 0.22	−6.16 ± 0.23	−6.15 ± 0.22	−6.15 ± 0.22	0.09 ± 0.04	0.685
	LE	−6.15 ± 0.24	−6.15 ± 0.24	−6.15 ± 0.25	−6.15 ± 0.24	−6.15 ± 0.24	0.08 ± 0.04	0.433
Posterior K2 (D)	RE	−6.50 ± 0.27	−6.49 ± 0.26	−6.49 ± 0.26	−6.49 ± 0.26	−6.49 ± 0.26	0.10 ± 0.04	0.623
	LE	−6.49 ± 0.29	−6.49 ± 0.28	−6.49 ± 0.27	−6.48 ± 0.27	−6.48 ± 0.28	0.11 ± 0.04	0.439
CCT (mm)	RE	539.82 ± 35.56	538.36 ± 25.82	537.64 ± 35.70	536.15 ± 35.51	535.93 ± 36.60	8.29 ± 4.24	<0.001 *
	LE	542.06 ± 36.64	540.91 ± 36.04	539.53 ± 37.79	537.80 ± 36.25	537.45 ± 36.75	8.24 ± 6.41	<0.001 *
MCT (mm)	RE	536.87 ± 35.52	535.38 ± 35.51	534.76 ± 35.19	532.60 ± 36.48	532.83 ± 36.59	9.04 ± 6.75	<0.001 *
	LE	538.57 ± 35.98	537.85 ± 35.50	536.00 ± 37.22	534.61 ± 35.99	534.38 ± 36.19	8.39 ± 6.20	<0.001 *
CV (mm^3^)	RE	61.08 ± 3.50	60.69 ± 3.47	60.63 ± 3.58	60.46 ± 3.48	60.39 ± 3.53	1.15 ± 0.47	<0.001 *
	LE	61.26 ± 3.50	60.96 ± 3.51	60.98 ± 3.53	60.69 ± 3.57	60.58 ± 3.48	1.03 ± 0.55	<0.001 *

Data are presented as mean ± standard deviation (SD). Amplitude was defined as the difference between the maximum and minimum values recorded across the five time points. *p*-values reflect comparisons among the five timepoints, using repeated-measures ANOVA for normally distributed variables and the Friedman test for not normally distributed variables. Values below 0.05 are marked with an asterisk (*). Abbreviations: RE = right eye; LE = left eye; IOP = intraocular pressure; IOPcc = corneal-compensated intraocular pressure; CCT = central corneal thickness; MCT = minimum corneal thickness; CV = corneal volume.

**Table 2:** Diurnal changes in corneal aberrations and *C_NORM_*.

		09:00	11:30	14:00	16:30	19:00	Amplitude	*p*-Value
Oblique Astigmatism (µm)	RE	−0.113 ± 0.616	−0.005 ± 0.645	−0.045 ± 0.622	−0.012 ± 0.654	−0.050 ± 0.653	0.360 ± 0.180	0.001 *
	LE	0.317 ± 0.762	0.316 ± 0.760	0.317 ± 0.752	0.327 ± 0.780	0.359 ± 0.709	0.348 ± 0.190	0.663
Defocus (µm)	RE	−0.020 ± 0.354	0.270 ± 0.020	0.011 ± 0.089	0.030 ± 0.036	0.026 ± 0.022	0.087 ± 0.363	0.445
	LE	0.003 ± 0.181	0.006 ± 0.162	0.28 ± 0.020	0.049 ± 0.168	0.027 ± 0.049	0.090 ± 0.377	0.226
Vertical Astigmatism (µm)	RE	−1.562 ± 1.454	−1.640 ± 1.350	−1.644 ± 1.341	−1.645 ± 1.315	−1.683 ± 1.351	0.478 ± 0.724	0.337
	LE	−1.752 ± 1.332	−1.740 ± 1.402	−1.741 ± 1.361	−1.783 ± 1.369	−1.822 ± 1.321	0.439 ± 0.438	0.478
Oblique Secondary Astigmatism (µm)	RE	−0.056 ± 0.095	−0.052 ± 0.106	−0.051 ± 0.103	−0.046 ± 0.090	−0.053 ± 0.101	0.116 ± 0.060	0.934
	LE	0.035 ± 0.090	0.035 ± 0.105	0.028 ± 0.084	0.017 ± 0.096	0.014 ± 0.101	0.123 ± 0.086	0.574
Spherical Aberration (µm)	RE	−0.568 ± 0.478	−0.482 ± 0.243	−0.505 ± −0.270	−0.506 ± 0.246	−0.481 ± 0.264	0.208 ± 0.370	0.006 *
	LE	−0.529 ± 0.282	−0.520 ± 0.285	−0.497 ± 0.264	−0.473 ± 0.262	−0.461 ± 0.293	0.187 ± 0.243	0.020 *
Vertical Secondary Astigmatism (µm)	RE	−0.010 ± 0.556	−0.081 ± 0.182	−0.038 ± 0.237	−0.077 ± 0.194	−0.092 ± 0.212	0.238 ± 0.517	0.026 *
	LE	−0.032 ± 0.250	−0.018 ± 0.269	−0.055 ± 0.193	−0.091 ± 0.238	−0.090 ± 0.240	0.218 ± 0.339	0.015 *
*C_NORM_*	RE	1.063 ± 0.191	1.066 ± 0.154	1.082 ± 0.165	1.114 ± 0.176	1.123 ± 0.163	0.228 ± 0.127	0.002 *
	LE	1.120 ± 0.159	1.093 ± 0.130	1.111 ± 0.155	1.169 ± 0.176	1.149 ± 0.166	0.232 ± 0.137	0.002 *

Data are presented as mean ± standard deviation (SD). Amplitude was defined as the difference between the maximum and minimum values recorded across the five time points. *p*-values reflect comparisons among the five timepoints, using repeated-measures ANOVA for normally distributed variables and the Friedman test for not normally distributed variables. Values below 0.05 are marked with an asterisk (*). Abbreviations: RE = right eye; LE = left eye; *C_NORM_* = pressure-normalized curvature coefficient.

## Data Availability

The data presented in this study are available on request from the corresponding author. The data are not publicly available due to privacy or ethical restrictions.

## Abbreviations

The following abbreviations are used in this manuscript:

IOP	Intraocular Pressure
CCT	Central Corneal Thickness
RE	Right Eye
LE	Left Eye
K1	Flattest Keratometry
K2	Steepest Keratometry
MCT	Minimum Corneal Thickness

## References

[1] Wang Y., Cao H. (2022). Corneal and scleral biomechanics in ophthalmic diseases: An updated review. Med. Nov. Technol. Devices.

[2] Norrby S., Hirnschall N., Nishi Y., Findl O. (2013). Fluctuations in corneal curvature limit predictability of intraocular lens power calculations. J. Cataract. Refract. Surg..

[3] Anders P., Anders L.M., Barbara A., Szentmary N., Langenbucher A., Gatzioufas Z. (2022). Intraocular lens power calculation in eyes with previous corneal refractive surgery. Ther. Adv. Ophthalmol..

[4] Hamilton K.E., Pye D.C., Aggarwala S., Evian S., Khosla J., Perera R. (2007). Diurnal variation of central corneal thickness and Goldmann applanation tonometry estimates of intraocular pressure. J. Glaucoma.

[5] Kida T., Liu J.H.K., Weinreb R.N. (2006). Effect of 24-hour corneal biomechanical changes on intraocular pressure measurement. Investig. Ophthalmol. Vis. Sci..

[6] Shen M., Wang J., Qu J., Xu S., Wang X., Fang H. (2008). Diurnal variation of ocular hysteresis, corneal thickness, and intraocular pressure. Optom. Vis. Sci..

[7] Du Toit R., Vega J.A., Fonn D., Simpson T. (2003). Diurnal variation of corneal sensitivity and thickness. Cornea.

[8] Barberán-Bernardos L., Ariza-Gracia M.Á., Piñero D.P. (2024). Corneal and intraocular pressure changes associated to the circadian rhythms: A narrative review. Int. J. Ophthalmol..

[9] Hon Y., Wan K., Chen G.Z., Lu S.H., Lam D.C.C., Lam A.K.C. (2016). Diurnal Variation of Corneal Tangent Modulus in Normal Chinese. Cornea.

[10] Biswas S., Biswas P. (2023). Relationship between Diurnal Variation in Intraocular Pressure and Central Corneal Power. Optom. Vis. Sci..

[11] Lau W., Pye D.C. (2012). Associations between diurnal changes in Goldmann tonometry, corneal geometry, and ocular response analyzer parameters. Cornea.

[12] Vera J., Redondo B., Molina R., Jiménez R. (2022). Effects of water drinking on corneal biomechanics: The association with intraocular pressure changes. Indian J. Ophthalmol..

[13] Jiménez R., Molina R., Redondo B., Vera J. (2020). Effects of caffeine intake on the biomechanical properties of the cornea: A placebo-controlled, double-blind, crossover pilot study in low caffeine consumers. Graefes Arch. Clin. Exp. Ophthalmol..

[14] Frifelt L.E.W., Subhi Y., Holm L.M., Singh A. (2022). Impact of tobacco use on corneal thickness and endothelial health: A systematic review with meta-analyses. Acta Ophthalmol..

[15] Barberán-Bernardos L., Ariza-Gracia M.Á., Piñero D.P. (2026). Diurnal variation in corneo-scleral morphology. J. Optom..

[16] Shankar H., Taranath D., Santhirathelagan C.T., Pesudovs K. (2008). Anterior segment biometry with the Pentacam: Comprehensive assessment of repeatability of automated measurements. J. Cataract. Refract. Surg..

[17] Read S.A., Collins M.J. (2009). Diurnal variation of corneal shape and thickness. Optom. Vis. Sci..

[18] do Carmo M.P. (2016). The Geometry of the Gauss Map. Differential Geometry of Curves and Surfaces.

[19] Lakshminarayanan V., Fleck A. (2011). Zernike polynomials: A guide. J. Mod. Opt..

[20] Gullstrand A., Southall J.P.C. (1962). Appendix: The schematic eye. Helmholtz’s Treatise on Physiological Optics.

[21] Chung C.W., Girard M.J.A., Jan N.J., Sigal I.A. (2016). Use and Misuse of Laplace’s Law in Ophthalmology. Investig. Ophthalmol. Vis. Sci..

[22] Siqveland L.M., Skjæveland S.M. (2021). Derivations of the Young-Laplace equation. Capillarity.

[23] Smith G., Pierscionek B.K. (1998). The optical structure of the lens and its contribution to the refractive status of the eye. Ophthalmic Physiol. Opt..

[24] Langenbucher A., Szentmáry N., Weisensee J., Wendelstein J., Cayless A., Menapace R., Hoffmann P. (2021). Prediction model for best focus, power, and spherical aberration of the cornea: Raytracing on a large dataset of OCT data. PLoS ONE.

[25] Hoehn R., Mirshahi A., Hoffmann E.M., Kottler U.B., Wild P.S., Laubert-Reh D., Pfeiffer N. (2013). Distribution of intraocular pressure and its association with ocular features and cardiovascular risk factors: The gutenberg health study. Ophthalmology.

[26] Wang Y.X., Xu L., Wei W.B., Jonas J.B. (2018). Intraocular pressure and its normal range adjusted for ocular and systemic parameters. The Beijing Eye Study 2011. PLoS ONE.

[27] Conover W.J. (1999). Practical Nonparametric Statistics.

[28] van den Heuvel E., Zhan Z. (2022). Myths About Linear and Monotonic Associations: Pearson’s r, Spearman’s ρ, and Kendall’s τ. Am. Stat..

[29] Seitz L., Gaitan D., Berkemeier C.M., Berger C.T., Recher M. (2023). Cluster analysis of flowcytometric immunophenotyping with extended T cell subsets in suspected immunodeficiency. Immun. Inflamm. Dis..

[30] Cremonini M. (2024). Data Visualization in R and Python.

[31] Park E., Cho M., Ki C.S. (2009). Correct use of repeated measures analysis of variance. Korean J. Lab. Med..

[32] Sheldon M.R., Fillyaw M.J., Thompson W.D. (1996). The use and interpretation of the Friedman test in the analysis of ordinal-scale data in repeated measures designs. Physiother. Res. Int..

[33] Giráldez-Fernández M.J., Díaz-Rey A., García-Resua C., Yebra-Pimentel-Vilar E. (2008). Diurnal variations of central and paracentral corneal thickness and curvature. Arch. Soc. Esp. Oftalmol..

[34] Read S.A., Collins M.J., Iskander D.R. (2008). Diurnal variation of axial length, intraocular pressure, and anterior eye biometrics. Investig. Ophthalmol. Vis. Sci..

[35] Burfield H.J., Patel N.B., Ostrin L.A. (2018). Ocular Biometric Diurnal Rhythms in Emmetropic and Myopic Adults. Investig. Ophthalmol. Vis. Sci..

[36] Mierdel P., Krinke H.E., Pollack K., Spoerl E. (2004). Diurnal fluctuation of higher order ocular aberrations: Correlation with intraocular pressure and corneal thickness. J. Refract. Surg..

[37] Read S.A., Collins M.J., Carney L.G. (2005). The diurnal variation of corneal topography and aberrations. Cornea.

[38] Redondo B., Vera J., Molina R., Jiménez R. (2020). Short-term effects of caffeine intake on anterior chamber angle and intraocular pressure in low caffeine consumers. Graefes Arch. Clin. Exp. Ophthalmol..

[39] Furlanetto R.L., Facio A.C., Hatanaka M., Susanna R. (2010). Correlation between central corneal thickness and intraocular pressure peak and fluctuation during the water drinking test in glaucoma patients. Clinics.

[40] Cankurtaran V., Tekin K. (2019). Cumulative Effects of Smoking and Diabetes Mellitus on Corneal Endothelial Cell Parameters. Cornea.

[41] Ilhan N., Ilhan O., Coskun M., Daglioglu M.C., Ayhan Tuzcu E., Kahraman H., Keskin U. (2016). Effects of Smoking on Central Corneal Thickness and the Corneal Endothelial Cell Layer in Otherwise Healthy Subjects. Eye Contact Lens.

[42] Tello A., Navarro P.A., Pedraza-Concha A., Villamizar S.J., Galvis V. (2025). Sleeping Behavior and Keratoconus: A Scoping Review. Cesk Slov. Oftalmol..

[43] Alevi D., Perry H.D., Wedel A., Rosenberg E., Alevi L., Donnenfeld E.D. (2017). Effect of Sleep Position on the Ocular Surface. Cornea.

